# Altered Serum Acylcarnitines Profile after a Prolonged Stay in Intensive Care

**DOI:** 10.3390/nu14051122

**Published:** 2022-03-07

**Authors:** Anne-Françoise Rousseau, Sarah Schmitz, Etienne Cavalier, Benoit Misset, François Boemer

**Affiliations:** 1 Intensive Care Department and Burn Centre, University Hospital of Liège, University of Liège, 4000 Liège, Belgium; sarah_schmitz86@hotmail.com (S.S.); benoit.misset@chuliege.be (B.M.); 2 Clinical Chemistry Department, University Hospital of Liège, University of Liège, 4000 Liège, Belgium; etienne.cavalier@chuliege.be; 3 Biochemical Genetics Lab, Department of Human Genetics, University Hospital of Liège, University of Liège, 4000 Liège, Belgium; f.boemer@chuliege.be

**Keywords:** carnitine, critical illness, survivors, mitochondrial dysfunction, catabolism, fatty acid metabolism

## Abstract

A stay in intensive care unit (ICU) exposes patients to a risk of carnitine deficiency. Moreover, acylated derivates of carnitine (acylcarnitines, AC) are biomarkers for metabolic mitochondrial dysfunction that have been linked to post-ICU disorders. This study aimed to describe the AC profile of survivors of a prolonged ICU stay (≥7 days). Survivors enrolled in our post-ICU clinic between September 2020 and July 2021 were included. Blood analysis was routinely performed during the days after ICU discharge, focusing on metabolic markers and including AC profile. Serum AC concentrations were determined by LC-MS/MS and were compared to the reference ranges (RR) established from serum samples of 50 non-hospitalized Belgian adults aged from 18 to 81 years. A total 162 patients (65.4% males, age 67 (58.7–73) years) survived an ICU stay of 9.7 (7.1–19.3) days and were evaluated 5 (3–8) days after discharge. Their AC profile was significantly different compared to RR, mostly in terms of short chain AC: the sum of C3, C4 and C5 derivates reached 1.36 (0.98–1.99) and 0.86 (0.66–0.99) µmol/L respectively (*p* < 0.001). Free carnitine (C0) concentration of survivors (46.06 (35.04–56.35) µmol/L) was similar to RR (43.64 (36.43–52.96) µmol/L) (*p* = 0.55). C0 below percentile 2.5 of RR was observed in 6/162 (3.7%) survivors. Their total AC/C0 ratio was 0.33 (0.22–0.42). A ratio above 0.4 was observed in 45/162 (27.8%) patients. In ICU survivors, carnitine deficiency was rare, but AC profile was altered and AC/C0 ratio was abnormal in more than 25%. The value of AC profile as a marker of post-ICU dysmetabolism needs further investigations.

## 1. Introduction

Carnitine, a naturally occurring amino acid derivative, is a low molecular weight molecule that exists as two enantiomers: D- and L-carnitine; however, only the L-isomer is physiologically active. Carnitine may be obtained from dietary sources (75%) and is synthesized endogenously in the body (25%), predominantly in the liver [1]. Carnitine plays a critical role in cellular energy metabolism and in maintenance of free coenzyme A availability. Its main function is the transport of long-chain fatty acids into mitochondria, where they will undergo subsequent β-oxidation. Carnitine also acts as a scavenger, binding acyl residues deriving from the intermediary metabolism of amino acids and facilitating their elimination. Both roles of carnitine result in its esterification into acylcarnitine derivatives. As such, the endogenous carnitine pool is comprised of L-carnitine and acylcarnitines (AC), in which L-carnitine is the major representative, such as the normal acylcarnitine to L-carnitine ratio is 0.25 [2]. A ratio in the blood exceeding 0.4 is thought to represent disturbed mitochondrial metabolism [2,3].

Carnitine deficiency, defined as a state of tissue or blood carnitine concentration below the requirement for normal organ function, can lead to hypoketotic hypoglycemia, muscle weakness, and cardiomyopathy. Rare primary carnitine deficiencies are related to inherited conditions with deficiencies of enzymes or transporters involved in the carnitine-acylcarnitine shuttle. Secondary carnitine deficiencies may occur in vegetarians, in some clinical situations or as consequences of some drugs therapies (i.e., zidovudine, cyclosporine). Critically ill patients are at risk of carnitine deficiency, due to clinical conditions, supports or treatments [4], such as prolonged undernutrition, prolonged total parenteral nutrition (commercial formulations do not contain carnitine), prolonged continuous renal replacement therapy (loss of carnitine in the effluent) or valproate treatment (carnitine used for urinary excretion of drug derivates) [3,5]. Reports about carnitine levels in critically ill patients are rare. According to an old study in an heterogenous cohort, carnitine depletion was observed in ICU in less than 5% of the patients suffering from malnutrition [6]. Some other conflicting results were reported in small older studies [7]. Nevertheless, carnitine supplementation is feasible, and even suggested by some experts in critically ill patients at risk of deficiency [4].

Besides carnitine deficiency, mitochondrial function is proven to be severely impaired in critically ill patients. It has been linked to increased morbidity and mortality during the stay in the intensive care unit (ICU) [8]. Moreover, animal data suggest that chronic muscle weakness after sepsis is underlined by mitochondrial dysfunction [9]. Impairment of mitochondrial β-oxidation of fatty acids induces accumulation of non-oxidized fatty acyl-coA esters into the mitochondria, which will be scavenged by conjugation with carnitine, for instance. These acyl-conjugates reach the blood, and some of them are excreted in the urine [10]. To the best of our knowledge, potential acylcarnitine profile alteration has never been described in the context of critical illness.

This observational study aimed to assess the incidence of carnitine deficiency and describe the acylcarnitines profile of patients who survived a prolonged ICU stay, by comparing them to a reference population.

## 2. Materials and Methods

### 2.1. Patients—Data Sources

Our 6-unit adult intensive care department is in a university hospital and includes 44 beds. It also includes 6 beds in a dedicated burn intensive care unit. For some years now, patients surviving an ICU stay ≥7 days are routinely invited to our post-intensive care follow-up, excepting in cases of end-of-life condition, coma, known dementia or bedridden status. Additionally, they were not included if they were unable to communicate in French, the local language. The follow-up begins in the ward, during the first 7 days following ICU discharge: A nurse-led face-to-face standardized visit allows a first screening of mental and cognitive disorders using validated questionnaires. Physical performances assessment is performed by physiotherapists. At that time, a blood analysis is also performed, focusing on inflammation, nutritional, and metabolic biomarkers. In this context, measurement of acylcarnitine profile, triglycerides (TG), total cholesterol and C-reactive protein (CRP) is part of our standard analysis.

We defined a prolonged ICU stay as a stay exceeding percentile 50 or 75 of the common reported duration of ICU stay in our hospital and in France, or in Europe, respectively [11,12].

All consecutive patients discharged from ICU after a stay ≥7 days between 10 September 2020 and 7 July 2021 were included in the follow-up trajectory if they did not present the above described exclusion criteria. Clinical data and biological parameters were prospectively recorded after the first visit in the ward, during the first 7 days following ICU discharge. Demographic data and data related to the ICU stay were collected retrospectively and extracted from the medical charts.

In accordance with Belgian law, informed consent was not required because the study did not modify patients’ management and the data were anonymously collected. This interpretation was confirmed by the Ethics Committee of the University Hospital of Liege (Chair: Pr Vincent Seutin, local reference 2020/424).

### 2.2. Serum Acylcarnitine Profiling

The biological data were generated from one single laboratory (Unilab, CHU de Liège) accredited according to ISO 15.189 guidelines.

In the text, Cx refers to the number of carbons in the acyl chain of carnitine derivatives (for example, if x = 2, it refers to 2 carbons in the acyl chain), and the various classes of ACs are referred to as follows: (1) acetylcarnitine (C2) and short-chain ACs (SCACs: C3 + C4 + C5), (2) medium-chain ACs (MCACs: C6 + C8 + C10 + C12), (3) long-chain ACs (LCACs: C14 + C16 + C18) ACs, (4) hydroxylated ACs (OH-ACs), and (5) dicarboxylic ACs (DC-ACs).

Blood samples were collected through a central or peripheral venous line placed for clinical use, or through venous punction. Blood was drawn into a serum gel tube (BD Vacutainer, Becton, Dickinson and Company, Franklin Lakes, NJ, USA), before being centrifuged (3500 rpm, 15 min, 4 °C). Supernatant was frozen at −20 °C and stored for later analysis.

Serum ACs concentrations (free carnitine (C0), C2-, C3-, C3DC + C4-OH-, C4-, C5-, C5:1-, C5DC + C6-OH-, C5-OH + C4-DC-, C6-, C6-DC-, C8-, C8:1-, C10-, C10:1-, C10:2-, C12-, C12:1-, C14-, C14:1-, C14:2-, C14-OH-, C16-, C16:1-, C16-OH + C17, C16-OH-, C18-, C18:1-, C18:2-, C18-OH-, C18:1-OH-, C18:2-OH-carnitine) were determined by flow-injection on a TQ5500 tandem mass spectrometer (Sciex, Framingham, MA, USA), using Neobase2 kit (PerkinElmer, Waltham, MA, USA) with slight modifications [13,14,15,16].

Reference ranges for all acylcarnitines were previously established in our same accredited lab, using the same flow-injection–mass spectrometry method, according to EP28 CLSI (Clinical and Laboratory Standards Institute) guidelines. The reference intervals were defined using 50 serum samples of apparently healthy (and non-hospitalized) individuals aged from 18 to 81 years (55 (43–62.5) years), including 46% males (23/50).

### 2.3. Ancillary Biochemical Parameters

The following variables were collected: serum CRP, TG and total cholesterol. The normal range is 0–5 mg/L for CRP, <175 mg/dL for TG in non-fasting condition, <190 mg/dL for total cholesterol (Alinity C, Abbott). The amino-acid profile was determined by liquid chromatography coupled to mass spectrometry (LCMS) using aTRAQ assay (Sciex, Framingham, MA, USA) [17].

### 2.4. Analysis

Statistical analysis was performed using Graphpad Prism (version 9.0 for Mac OSX, Graphpad Inc., San Diego, CA, USA). Qualitative parameters were expressed as counts and percentages. Normality was assessed using the Shapiro–Wilk test. As some datasets did not pass the normality test, results were expressed as medians with lower and upper quartiles (Q1–Q3) for quantitative parameters. Qualitative variables were described using count and percent. Comparisons between data were made using Mann–Whitney test, and using Fisher’s exact test for categorical variables. Correlations between results were tested using Spearman test. A *p* value <0.05 was considered statistically significant.

## 3. Results

**Figure 1:**
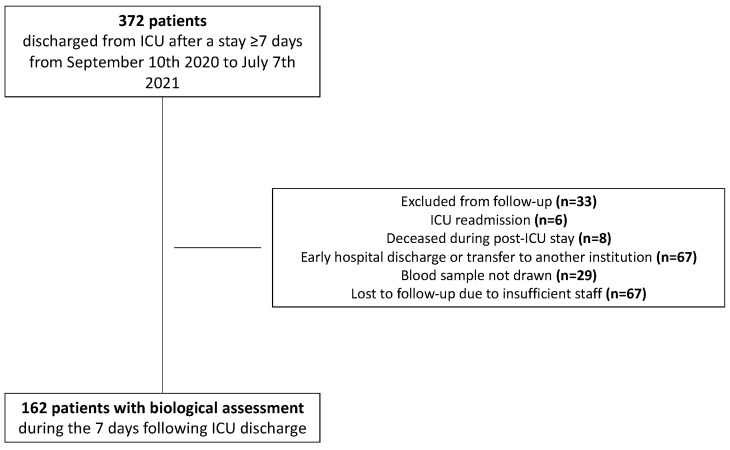
Flow chart.

From 10 September 2020, until 7 July 2021, 372 patients were discharged alive from ICU after a stay ≥7 days. Of these patients, 210 could not been evaluated in the first step of our post-ICU follow-up trajectory. Finally, 162 patients had the post-ICU follow-up blood analysis and were analyzed (Figure 1). Descriptive characteristics of the included subjects are detailed in Table 1. Blood samples were obtained 5 (3–8) days after ICU discharge.

**Figure 2:**
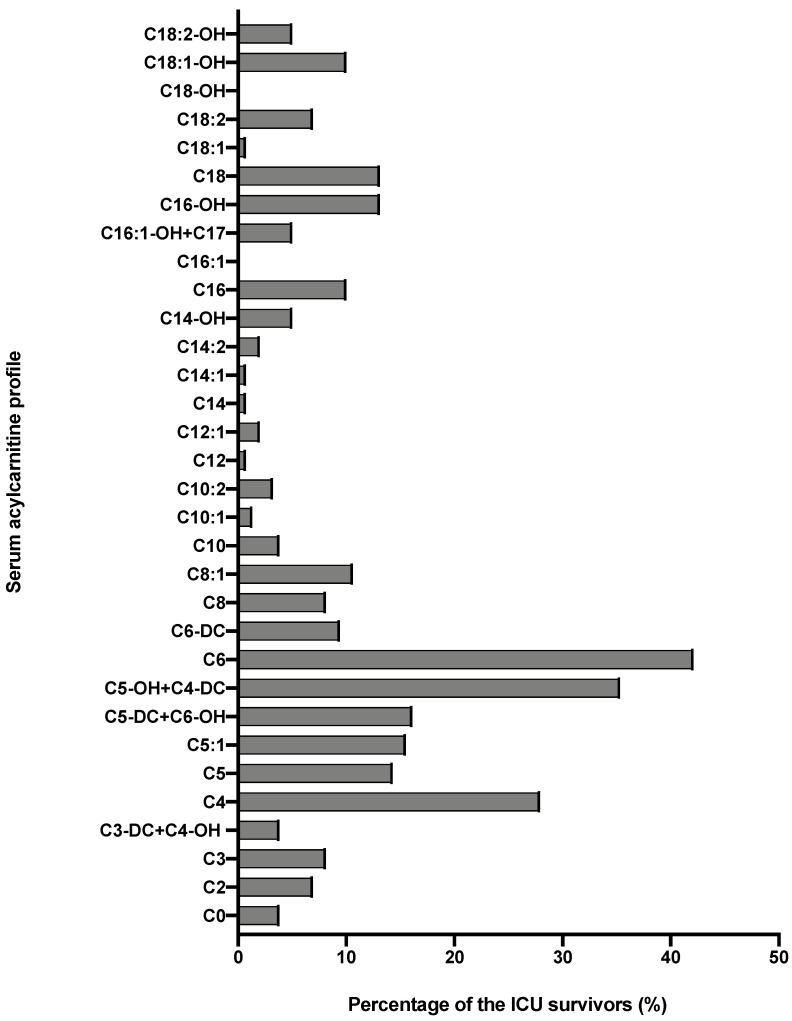
Proportion of ICU survivors with acylcarnitines concentrations above percentile 97.5 of the reference adult population.

AC profile of ICU survivors, compared to the reference ranges, is detailed in Table 2. The two profiles were different, mainly in terms of short-chain AC. The sum of short-chain AC (C3, C3-DC + C4-OH, C4, C5, C5:1, C5-OH + C4-DC) was significantly higher in ICU survivors than reference range: respectively, 1.36 (0.98–1.99) μmol/L and 0.86 (0.66–0.99) μmol/L (*p* < 0.001). The proportion of AC profile was also different in terms of C6- and C16-carnitines (Table 2). The proportion of patients with AC concentrations > percentile 97.5 of the reference population is described in Figure 2.

On the contrary, C0 (free carnitine) concentration was similar in ICU survivors was similar to references ranges (Table 2). Only 6/162 (3.7%) patients had a C0 concentration < percentile 2.5 of the reference population. No difference in C0 concentration was observed between patients with an ICU length of stay (LOS) ≥30 days, and in those with a shorter LOS: respectively, 43.57 (26.52–49.73) μmol/L and 47.18 (35.31–56.97) μmol/L (*p* = 0.153). Similarly, similar C0 concentrations were observed in patients who received parenteral nutrition during ICU stay (total or supplemental) and in those fed using alternative routes: respectively, 45.53 (35.13–52.83) μmol/L and 46.38 (34.89–57.05) μmol/L (*p* = 0.753). There was no difference in C0 concentration between medical patients and surgical patients: respectively, 45.34 (32.0–53.87) μmol/L and 44.43 (37.17–57.72) μmol/L (*p* = 0.065). On the contrary, patients who benefited from a continuous veno-venous hemofiltration (CVVH) had lower C0 concentrations after ICU discharge than patients without renal replacement therapy: respectively, 30.93 (26.91–52.97) μmol/L and 46.94 (36.31–56.40) μmol/L. This difference did not reach statistical significance (*p* = 0.06), and the proportion of C0 deficiency in both subgroups was similar (*p* = 0.314).

The AC/C0 ratio was 0.32 (0.22–0.42), significantly higher than in the reference population (0.25 (0.17–0.35)) (*p* = 0.012). A ratio >0.4 was observed in 45/162 (27.8%) patients. The ratio was significantly higher in patients who received propofol than in patients who did not receive propofol: respectively, 0.33 (0.25–0.44) vs. 0.26 (0.17–0.39) (*p* = 0.018). On the contrary, the ratio was similar in patients who benefited from mechanically ventilated and in those who were not mechanically ventilated: respectively, 0.32 (0.22–0.43) vs. 0.33 (0.22–0.41) (*p* = 0.997). The severity of critical illness did not influence C0 concentrations: similar C0 concentrations were observed in patients with severity scores lower than the median score, compared to more severely ill patients: respectively, 0.27 (0.2–0.41) vs. 0.36 (0.26–0.47) according to SOFA score (*p* = 0.111), and 0.36 (0.22–0.48) vs. 0.36 (0.22–0.42) according to SAPS 2 score (*p* = 0.681).

The other blood biomarkers measured at the same time of AC are described in Table 3. No correlation was found between TG or total cholesterol concentrations and the AC/C0 ratio. On the contrary, this ratio was weakly but significantly correlated with CRP: r_s_ = 0.33 (95% confidence interval 0.18 to 0.46) (*p* < 0.001). The sum of short-chain AC was weakly correlated to the sum of branched chains amino-acids (leucine, isoleucine and valine, BCAA): r = 0.18 (95% confidence interval 0.01–0.34), *p* = 0.03, but not with glutamine, phenylalanine nor tyrosine. There was no correlation between C3 and methionine.

## 4. Discussion

This study is the first to describe the AC profile in a general population of survivors of a prolonged ICU stay. Unlike suspected, carnitine deficiency was rare, observed in less than 5% of the patients. On another hand, their AC profile was different from a reference adult population in terms of short-chain AC and AC/C0 ratio.

Carnitine deficiency was not prevalent in the present cohort. There was no association between C0 concentration and described risk factors for carnitine deficiency in chronic critical illness (i.e., extremely long ICU stay, nutritional support by parenteral route, or CVVH) [4]. Carnitine supplementation does not seem to be indicated in this isolated context, based on blood levels.

Short-chain AC were found in excess in the present cohort, compared to the reference population. Short-chain AC can be derivates from alternative energy sources, such as BCAA [3,18]. Systemic inflammation, as observed in sepsis and trauma, induces catabolism, and also a leucine release from visceral tissues, leading to an increase in BCAA, prompting the utilization of carnitine and the production of C3, C4 and C5 derivates [18,19]. In the present study, we found a weak correlation between short-chain AC concentration and BCAA concentration, reinforcing this hypothesis. From this point of view, AC profile could be a biomarker of catabolism. Recognition of the transition between catabolic and anabolic phase following critical illness is of great interest, as it can trigger pharmacological strategies or changes in physical exercises and nutritional strategies [20]. Urea-to-creatinine ratio and the creatinine-to-cystatin C ratio (known as the sarcopenia index) are both potential biomarkers of catabolism, as they were correlated to muscle mass [21]. However, they are not suitable for assessment in case of concomitant acute kidney injury or renal replacement therapy, two situations frequently encountered in the ICU and post-ICU context. There is currently no evidence for AC profile as a marker of catabolism in critical illness context, but, undeniably, this should be further explored.

An increased AC/C0 ratio was observed in more than 25% of the present cohort. An AC/C0 ratio in the blood exceeding 0.4 is thought to reflect disturbed mitochondrial metabolism [2,3]. Mitochondrial dysfunction is observed as early as acute phase of a critical illness, at least in the muscles [22]. This mitochondrial dysfunction has been linked to the severity of illness and related mortality [23,24]. Recent data in an animal model of sepsis suggested that mitochondrial dysfunction is sustained in survivors [9]. In the context of critical illness, inflammation and propofol are two well-known triggers for mitochondrial dysfunction [8,25]: this is corroborated in the present study where CRP concentrations correlated with AC/C0 ratio, and where this ratio was significantly higher in patients who received a propofol-based sedation. Investigating the mitochondrial function is not easy in clinical practice, and the AC/C0 ratio could be a non-invasive and simple method, if further proved against reference or other validated methods, such as skin or muscle biopsy or proteomic analysis [26]. Indeed, the monitoring of mitochondrial function will be of interest in the near future, as mitochondrial therapies are increasingly developed and/or tested for more than a decade: antioxidants, reactive oxygen species or mitochondrial membrane stabilizers could have beneficial impacts on organ dysfunction and survival in sepsis [27].

This study surely extends the knowledge regarding AC profile and metabolic disorders in critically ill patients. Measurement of AC profile was performed using a reference method (mass spectrometry), allowing the measurement of C0 and the entire range of its acyl-esters. Using similar analytical method, similar C0 and AC concentrations have been previously reported in the literature [28]. However, some limitations need to be acknowledged. First, no sample size was calculated, as it was the very first report of AC profile in ICU survivors. Second, the studied population was older and consisted of more males than the reference population. Impact of age on carnitine concentrations is controversial [28,29]. Gender has been supposed to influence carnitine concentrations [28], but no differences in C0 and AC concentrations between males and females were observed in the reference population as in the ICU survivors. Third, this study is a retrospective analysis of prospectively recorded data. It would have been interesting to concomitantly investigate functional outcome such as muscle strength or assess the link between AC profile and mid-term mortality or hospital readmission rates. Unfortunately, such data were not available for the present cohort. Fourth, this is a cross-sectional study without longitudinal assessment of the AC profile. A further study should focus on the spontaneous evolution of the observed alterations in AC profile: this work is ongoing in our follow-up clinic. Finally, it would have been informative to have pre-ICU values. Such evaluation is, by definition, challenging as ICU admissions are usually unexpected. Thus, baseline biological status is generally not assessable.

## 5. Conclusions

In the present cohort of ICU survivors discharged after a prolonged stay, carnitine deficiency was observed in less than 5% of the patients. On the contrary, they exhibited different AC profile in terms of short-chain AC compared to the reference values, and the AC/C0 ratio was abnormally increased in more than 25% of the patients. The role of AC profile as catabolism or mitochondrial dysfunction biomarker in the context of critical illness and post-ICU trajectory should be further explored, in view of the potential clinical implications.

## Figures and Tables

**Table 1:** Cohort demographics.

Data		n = 162
Age, y		67 (58.7–73)
Male, n (%)		106 (65.4)
Weight, kg		75.6 (64–90.5)
Height, cm		170 (163–177)
BMI, kg/m^2^		26.5 (22.8–30.1)
Comorbidities, n (%)	HIV	1 (0.6)
	Epilepsy treated by valproate	3 (18.5)
Admission category, n (%)	Medical	83 (51.2)
	Surgical	79 (48.8)
Primary failure, n (%)	Cardiovascular	66 (40.7)
	Pulmonary	31 (19.1)
	Neurologic	29 (17.9)
	Digestive	8 (4.9)
	Hepatic	4 (2.5)
	Polytrauma	5 (3.1)
	Other	19 (11.7)
SAPS II		50.5 (32–72)
Mechanical ventilation >24 h, n (%)		91 (56.2)
Duration of mechanical ventilation, d		6 (2–14)
Renal replacement therapy, n (%)		10 (6.2)
Duration of renal replacement therapy, d		8 (6.5–12.2)
Extracorporeal membrane oxygenation, n (%)		2 (1.2)
Propofol-based sedation, n (%)		113 (69.8)
Duration of propofol infusion, d		4 (2–8)
Valproate treatment during ICU stay, n (%)		4 (2.5)
Oral nutrition, n (%)		70 (43.2)
Enteral nutrition, n (%)		97 (59.9)
Duration of enteral nutrition, d		9 (6–17)
Parenteral nutrition, n (%)		23 (14.2)
Duration of parenteral nutrition, d		7 (4–9)
ICU LOS, d		9.7 (7.1–19.3)
Hospital LOS, d		35 (22–57)

Data are expressed as medians with lower and upper quartiles (Q1–Q3). BMI: body mass index; HIV: human immunodeficiency virus; ICU: intensive care unit, LOS: length of stay; SAPS II: Simplified Acute Physiology Score II.

**Table 2:** Acylcarnitines concentration in ICU survivors and in the reference population.

Acylcarnitines (μmol/L)	ICU Survivors n = 162	Reference Ranges, Based on 50 Serum Samples of Apparently Healthy Individuals	*p* Value
C0	46.06 (35.04–56.35)	43.64 (36.43–52.96)	0.549
C2	9.92 (6.96–15.12)	9.92 (5.45–11.46)	0.058
C3	0.81 (0.53–1.20)	0.41 (0.30–0.48)	<0.001
C3-DC + C4-OH	0.04 (0.02–0.08)	0.03 (0.02–0.05)	0.005
C4	0.28 (0.18–0.42)	0.15 (0.12–0.22)	<0.001
C5	0.11 (0.07–0.15)	0.10 (0.07–0.13)	0.353
C5:1	0.01 (0.01–0.02)	0.01 (0.01–0.02)	0.590
C5-DC + C6-OH	0.14 (0.08–0.21)	0.08 (0.06–0.11)	<0.001
C5-OH + C4-DC	0.04 (0.03–0.07)	0.02 (0.02–0.03)	<0.001
C6	0.12 (0.07–0.32)	0.05 (0.04–0.07)	<0.001
C6-DC	0.08 (0.05–0.17)	0.06 (0.04–0.09)	0.031
C8	0.11 (0.07–0.17)	0.10 (0.07–0.14)	0.315
C8:1	0.15 (0.11–0.23)	0.14 (0.09–0.21)	0.044
C10	0.14 (0.09–0.24)	0.16 (0.10–0.23)	0.459
C10:1	0.08 (0.05–0.12)	0.07 (0.05–0.10)	0.528
C10:2	0.02 (0.01–0.03)	0.01 (0.01–0.02)	0.037
C12	0.05 (0.03–0.07)	0.06 (0.04–0.09)	0.022
C12:1	0.07 (0.04–0.12)	0.07 (0.04–0.12)	0.708
C14	0.03 (0.02–0.04)	0.03 (0.02–0.05)	0.474
C14:1	0.08 (0.04–0.11)	0.03 (0.05–0.14)	0.412
C14:2	0.02 (0.01–0.04)	0.02 (0.02–0.04)	0.458
C14-OH	0.01 (0.01–0.01)	0.01 (0.01–0.01)	0.33
C16	0.17 (0.12–0.23)	0.13 (0.10–0.18)	0.003
C16:1	0.03 (0.02–0.05)	0.03 (0.02–0.05)	0.928
C16-OH + C17	0.01 (0.01–0.01) P2.5 = 0.002 P97.5 = 0.030	0.01 (0.01–0.01) P2.5 = 0.002 P97.5 = 0.068	0.006
C16-OH	0.01 (0.01–0.01) P2.5 = 0.000 P97.5 = 0.029	0 (0–0) P2.5 = 0.001 P97.5 = 0.023	0.013
C18	0.04 (0.03–0.06)	0.04 (0.03–0.06)	0.436
C18:1	0.17 (0.11–0.25)	0.16 (0.10–0.23)	0.384
C18:2	0.05 (0.03–0.06)	0.04 (0.03–0.06)	0.486
C18-OH	0.01 (0.01–0.01)	0 (0–0)	0.945
C18:1-OH	0.01 (0.01–0.01)	0 (0–0)	0.971
C18:2-OH	0.01 (0.01–0.01)	0 (0–0)	0.245

Data are expressed as mean and [IQR]. For some very low results, P2.5 and P97.5 are also detailed.

**Table 3:** Ancillary biochemical parameters.

Biomarkers	ICU Survivors n = 162	Reference Ranges Provided by the Manufacturers
C-reactive protein (mg/L)	35.9 (15.1–82.2)	0–5
Triglycerides (mg/dL)	136 (104.5–180)	<175
Total cholesterol (mg/dL)	139 (113.5–167.5)	<190
Leucine (μmol/L)	129.5 (106.5–167.5)	73.5–228
Isoleucine (μmol/L)	80.3 (63.5–103.8)	36.5–132
Valine (μmol/L)	211 (174–259.8)	105–352
Glutamine (μmol/L)	486.5 (430–589.5)	311–650
Methionine (μmol/L)	21.95 (17.48–29.13)	13.1–34.1
Phenylalanine (μmol/L)	78.85 (61.15–101)	41.3–130
Tyrosine (μmol/L)	55.2 (46.05–71.68)	37.6–101

Data are expressed as mean and (IQR).

## Data Availability

The datasets used and/or analyzed during the current study are available from the corresponding author on reasonable request.
